# Surgical Treatment for Pulmonary Aspergillosis: A Thirty-Year Single-Center Experience and the Contemporary Role of Surgery

**DOI:** 10.5761/atcs.oa.26-00151

**Published:** 2026-09-18

**Authors:** Takuro Miyazaki, Takahiro Takazono, Ryoichiro Doi, Koichiro Shimoyama, Tomohiro Obata, Takaaki Nakatsukasa, Hiromi Ichikawa, Aya Itakura, Ryosuke Ogata, Keitaro Matsumoto

**Affiliations:** 1 Division of Thoracic and Pelvic Surgery, Department of Surgery, Nagasaki University Graduate School of Biomedical Sciences, Nagasaki, Nagasaki, Japan; 2 Department of Respiratory Medicine, Nagasaki University Hospital, Nagasaki, Nagasaki, Japan

**Keywords:** pulmonary aspergillosis, pulmonary resection, surgery, bronchial artery embolization

## Abstract

**Purpose:**

Surgical treatment for pulmonary aspergillosis remains technically demanding because of dense pleural adhesions, chronic inflammation, and the risk of major bleeding. We evaluated temporal changes in patient characteristics, perioperative management, and surgical outcomes over a 30-year period at a single institution.

**Methods:**

We retrospectively reviewed 57 consecutive patients who underwent surgery for pulmonary aspergillosis between 1992 and 2023. Patients were divided into an early era (1992–2007, n = 23) and a late era (2008–2023, n = 34). Clinicopathological characteristics, operative variables, perioperative management, and postoperative outcomes were compared.

**Results:**

Hemoptysis or bloody sputum and previous pulmonary tuberculosis were significantly less frequent in the late era. Lobectomy was the most common procedure, while video-assisted thoracoscopic surgery was introduced during the later period. Operative time (350 vs. 250 min, p = 0.016) and median blood loss (750 vs. 178 mL, p = 0.003) were significantly reduced in the late era. However, the incidence of major postoperative complications and overall survival did not differ significantly between the 2 eras.

**Conclusion:**

Surgical management of pulmonary aspergillosis has evolved with improvements in operative efficiency and blood loss while maintaining acceptable perioperative and long-term outcomes. Careful patient selection and multidisciplinary perioperative management remain essential, particularly for patients with chronic pulmonary aspergillosis.

## Introduction

Pulmonary aspergillosis remains a challenging infectious disease in thoracic surgery despite advances in antifungal therapy and perioperative management. Chronic pulmonary aspergillosis (CPA) and simple pulmonary aspergilloma (SPA) frequently develop in patients with underlying structural lung diseases, including prior pulmonary tuberculosis, chronic obstructive pulmonary disease, and bronchiectasis.^1–3)^ These patients often present with hemoptysis, recurrent pulmonary infection, and progressive respiratory impairment, occasionally resulting in life-threatening conditions requiring surgical intervention.

Although antifungal therapy^4,5)^ has evolved substantially with the introduction of triazole agents such as voriconazole and itraconazole, medical treatment alone is often insufficient for localized disease, uncontrolled hemoptysis, or refractory infection. Surgical resection therefore continues to play an important role in selected patients as a potentially curative treatment and an effective strategy for symptom control. However, surgery for pulmonary aspergillosis remains technically demanding because dense pleural adhesions, destroyed lung parenchyma, and developed collateral vessels frequently result in prolonged operative time, massive bleeding, and high perioperative morbidity.

Previous studies have reported perioperative mortality rates ranging from 4% to 15%, particularly in patients with CPA.^6–14)^ In addition, patient backgrounds and treatment strategies for pulmonary aspergillosis have changed considerably over recent decades. The prevalence of post-tuberculosis destroyed lung has decreased in developed countries, while the use of modern antifungal agents, bronchial artery embolization (BAE), advanced surgical energy devices, and a minimally invasive procedure called video-assisted thoracoscopic surgery (VATS) has expanded.^15,16)^ In recent years, uniportal VATS^17–19)^ and robotic-assisted thoracoscopic surgery (RATS)^20)^ have been attempted for selected cases. Nevertheless, the impact of these temporal changes on surgical outcomes and the contemporary role of surgery remain incompletely understood.

The present study retrospectively reviewed a 30-year single-institution experience of surgical treatment for pulmonary aspergillosis. We aimed to evaluate temporal changes in patient characteristics, perioperative management, and surgical outcomes, and to clarify the current role and limitations of surgery in the modern antifungal era.

## Patients and Methods

### Patients

We retrospectively reviewed consecutive patients who underwent surgical treatment for pulmonary aspergillosis at Nagasaki University Hospital between January 1992 and December 2023. A total of 57 patients were included in this study. The diagnosis of pulmonary aspergillosis was established based on clinical findings, radiological findings, microbiological examinations, pathological findings, and multidisciplinary clinical assessment.^5)^

**Fig. 1:**
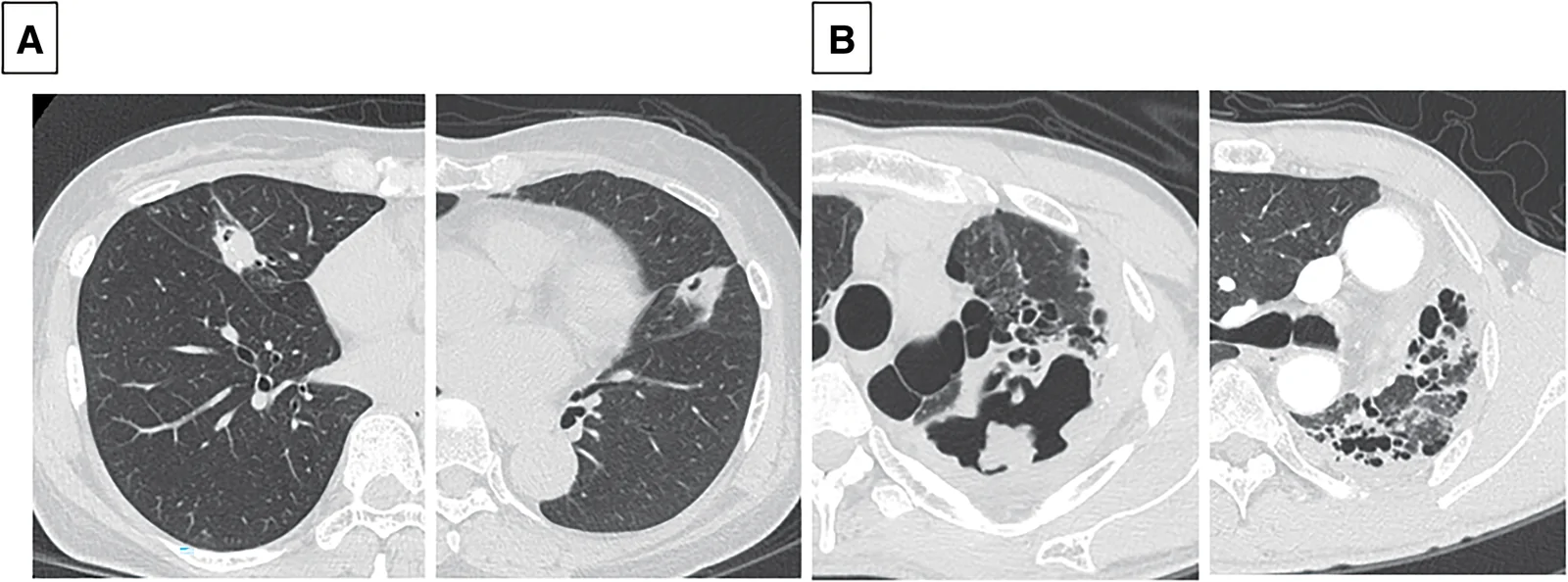
Radiological classification of pulmonary aspergillosis. (**A**) Simple pulmonary aspergillosis (SPA). (**B**) Chronic pulmonary aspergillosis (CPA).

According to established radiological criteria and current clinical guidelines for CPA,^1–3)^ patients were classified into 2 disease subtypes in this study: CPA and SPA. SPA was defined as a solitary fungal ball within a preexisting lung cavity without significant surrounding parenchymal destruction or progression over time, whereas CPA was defined as progressive cavitary pulmonary disease with surrounding inflammatory destruction, pleural thickening, or multiple cavities associated with chronic symptoms and radiological progression (**Fig. 1**).

To evaluate temporal changes in surgical strategy and perioperative outcomes, the study period was divided into 2 eras (1992–2007 and 2008–2023). The year 2008 was selected as the cutoff based on clinically relevant changes in the management of pulmonary aspergillosis at our institution, including the wider use of modern triazole antifungal agents such as voriconazole and the gradual introduction of minimally invasive thoracic surgery. Importantly, this cutoff was determined based on these changes in clinical practice rather than on observed differences in surgical outcomes.

Patient demographics and the following clinicopathological factors were recorded: as patient factors, age, sex, body mass index (BMI: kg/m^2^), a history of pulmonary tuberculosis, the type of pulmonary aspergillosis determined by chest computed tomography (CT), any symptom related to pulmonary aspergillosis, and perioperative antifungal therapy. As surgical factors, preoperative BAE, surgical procedures (lobectomy, limited resection, and others), operation time, bleeding, surgical complications, and long-term survival were retrospectively analyzed and compared between the 2 groups.

This study was approved by the institutional review board of Nagasaki University Hospital (Number:24091203-2). The requirement for informed consent was waived because of the retrospective nature of the study.

### Preoperative management and surgical indications

Surgical indications were determined through multidisciplinary discussion involving thoracic surgeons, infectious disease specialists, pulmonologists, and radiologists. Surgery was considered in patients with SPA and localized disease associated with recurrent hemoptysis, persistent bloody sputum, recurrent pulmonary infection, progression despite antifungal therapy, or difficulty in controlling symptoms medically. In selected patients with active hemoptysis, preoperative BAE was performed to stabilize the respiratory condition and reduce the risk of intraoperative bleeding.

Antifungal therapy, including triazole agents such as itraconazole and voriconazole, was administered according to disease activity and at the discretion of infectious disease specialists. Since the late 2000s, modern azole-based antifungal therapy became widely available and was increasingly incorporated into perioperative management. Whenever possible, we attempted to identify fungal species from sputum, bronchoalveolar lavage fluid (BALF), and resected lung specimens, and to perform antifungal susceptibility testing.

### Surgical procedures

The extent of resection was determined according to disease localization, pulmonary function, and intraoperative findings. Lobectomy was the preferred standard procedure for localized lesions whenever feasible. Sublobar resection, including segmentectomy or wedge resection, was selected in patients with limited disease or poor pulmonary reserve. Completion pneumonectomy was performed in selected patients with extensive destroyed lungs. In cases of pneumonectomy, the bronchial stump was covered with latissimus dorsi, serratus anterior, or intercostal muscle flaps. In cases considered unsuitable for anatomical resection for various reasons, cavernostomy or muscle flap plombage procedures were performed in the early era.

VATS via a 4-portal approach without a rib spreader has been established at our institution since around 2008 and was applied in selected patients without extensive hilar invasion or severe pleural adhesions. Postoperative complications were assessed according to the Clavien–Dindo classification.^21)^

Postoperative follow-up schedules were determined by the treating surgeons. In general, patients were followed up at 1- to 3-month intervals by infectious disease specialists after the first postoperative visit to the surgical outpatient clinic and were evaluated by routine laboratory tests and chest CT. Postoperative antifungal therapy was administered based on the clinical judgment of infectious disease specialists and pulmonologists, considering the patient’s postoperative course and the extent of residual lesions. Recently, a comprehensive review of pulmonary aspergilloma and its clinical management has been published,^4)^ which will serve as a guideline for clinicians, and we intend to implement management practices in accordance with it.

### Statistical analysis

The differences in clinicopathological factors in each period were evaluated by the chi-squared test, paired *t*-test, or Mann-Whitney U test, depending on the type of data. Overall survival (OS) was defined as the time interval from the date of surgery to death, and disease-specific survival (DSS) was defined as the time interval from the date of surgery to death due to aspergillosis or surgical mortality; these were analyzed using the Kaplan–Meier method and the log-rank test. Univariate and multivariate survival analyses were performed with a Cox proportional hazards regression model. p Values less than 0.05 were considered significant. JMP 19 (SAS Institute Inc., Cary, NC, USA) was used for all statistical analyses.

## Results

A total of 57 patients underwent surgical treatment for pulmonary aspergillosis during the study period, including 23 patients in the early era (1992–2007) and 34 patients in the late era (2008–2023). Patient characteristics are summarized in **Table 1**.

**Table 1:** Characteristics of surgically treated patients with pulmonary aspergillosis

Variables		Early (n = 23)	Late (n = 34)	p Value
Patient factors				
Age	Year, median, IQR	63 (51–67)	66 (55–71)	0.1407
Sex	Male/Female	15/8	26/8	0.3831
BMI	kg/m^2^, median, IQR	19.7 (17.3–22.7)	20.0 (17.7–22.8)	0.6843
COPD + IP	Yes/No	4/19	17/17	0.0142*
Cardiovascular and renal diseases	Yes/No	1/22	7/27	0.0833
Immunosuppressant use	Yes/No	3/20	8/26	0.4966
History of pulmonary tuberculosis	Yes/No	16/7	6/28	0.0001*
Symptoms	Yes/No	22/1	21/13	0.0041*
Type of PA	SPA/CPA/Others	8/13/2	16/15/3	0.6310
Preoperative BAE	Yes/No	7/16	5/29	0.1935
Antifungal therapy before surgery	Yes/No	18/5	18/16	0.0919
Surgical factor				
Procedure	Lobectomy/Limited/Others	19/2/2	29/3/2	0.9213
VATS	Yes/No	1/22	6/28	0.2227
Operation time	min, median, IQR	350 (230–456)	250 (169–324)	0.0161*
Blood loss	mL, median, IQR	750 (410–1570)	178 (60–560)	0.003*
Postoperative complications	Yes/No	9/14	12/22	0.7866
Grade ≥III complications	Yes/No	7/16	8/26	0.7600
Length of postoperative stay	days, median, IQR	18 (12–31)	10 (8–15)	0.0004*
Antifungal therapy after surgery	Yes/No	16/7	21/13	0.5847

*Statistically significant difference.

BMI, body mass index; BAE, bronchial artery embolization; COPD, chronic obstructive pulmonary disease; IP, interstitial pneumonia; PA, pulmonary aspergillosis; VATS, video-assisted thoracoscopic surgery; IQR, interquartile range; SPA, simple pulmonary aspergilloma; CPA, chronic pulmonary aspergillosis

Median age was 63 years (interquartile range [IQR], 51–67) in the early era and 66 years (IQR, 55–71) in the late era (p = 0.1407). Male patients accounted for 65% and 76% of cases, respectively (p = 0.3831). Patients with respiratory disease, including chronic obstructive pulmonary disease and interstitial pneumonia, were more common in the late era (p = 0.0142). A history of pulmonary tuberculosis significantly decreased from 70% in the early era to 18% in the late era (p = 0.0001). Symptomatic presentation also significantly decreased over time (96% vs. 62%, p = 0.0041). Disease subtype distribution was similar between the 2 groups, with SPA observed in 8 and 16 patients and CPA in 13 and 15 patients in the early and late eras, respectively (p = 0.631).

Preoperative BAE was performed in 12 patients (21%), including 7 patients in the early era and 5 in the late era (p = 0.1935). Preoperative antifungal therapy was administered in 18 patients (78%) in the early era and 18 patients (53%) in the late era (p = 0.0919).

Lobectomy was the most common surgical procedure, performed in 48 patients, whereas limited resection was performed in 5 patients and other procedures, including cavernostomy or muscle plombage, in 4 patients. VATS was introduced around the late era and performed in 7 patients overall.

Perioperative outcomes improved significantly in the late era. The median operation time decreased from 350 min (IQR, 230–456) to 250 min (IQR, 169–324) (p = 0.0161), and the median intraoperative blood loss decreased from 750 mL (IQR, 410–1570) to 178 mL (IQR, 60–560) (p = 0.003). The median postoperative hospital stay also significantly decreased from 18 days (IQR, 12–31) to 10 days (IQR, 8–15) (p = 0.0004).

Patients who underwent preoperative BAE showed significantly greater intraoperative blood loss (1050 vs. 270 mL, p = 0.0038) and longer operation time (397 vs. 262 min, p = 0.0119) than those without BAE, reflecting the higher proportion of CPA cases in the BAE group (83% vs. 40%, p = 0.0261) (**Table 2**).

**Table 2:** Outcome of bronchial artery embolization (BAE)

		BAE (+) (n = 12)	BAE (−) (n = 45)	p Value
Period	Early/Late	7/ 5	16/29	0.1935
Blood loss	mL, median, IQR	1050 (470–2289)	270 (90–750)	0.0038*
Operation time	min, median, IQR	397 (257–538)	262 (207–345)	0.0119*
Type of PA	(SPA/CPA/Others)	2/10/0	22/18/5	0.0261*

*Statistically significant difference.

PA, pulmonary aspergillosis; IQR, interquartile range; SPA, simple pulmonary aspergilloma; CPA, chronic pulmonary aspergillosis

Postoperative complications occurred in 9 patients (39%) in the early era and 12 patients (35%) in the late era (p = 0.7866). Severe complications of Clavien–Dindo grade III or higher occurred in 7 and 8 patients, respectively (p = 0.7600). Major complications included bronchopleural fistula, pyothorax, prolonged air leak, postoperative pneumonia, acute exacerbation of interstitial pneumonia, and postoperative bleeding requiring reoperation (**Table 3**). Surgery-related mortality occurred in 2 patients (3.5%). Furthermore, logistic regression analysis was performed using patient background factors, surgical factors, and differences in time periods to predict complications of grade 3 or higher, but no significant factors were detected (data not shown).

**Table 3:** Postoperative complications according to the Clavien-Dindo classification in patients with pulmonary aspergillosis

Complications	Early (n = 9)		Complications	Late (n = 12)	
	Grade	Cases		Grade	Cases
BPF → fenestration	V	1	AE of interstitial pneumonia	V	1
Pyothorax → fenestration	IV	1	BPF → fenestration	IV	1
Pyothorax → redo thoracotomy	IV	1	Pyothorax → fenestration	IV	1
Drug-induced pneumonia	IV	1	Bleeding → redo thoracotomy	IV	1
Pneumonia	IV	1	Hemoptysis during induction of anesthesia	IV	1
Prolonged air leak (>7 days)	III	2	AE of interstitial pneumonia	III	1
Atrial fibrillation	II	1	Prolonged air leak (>7 days)	III	1
Wound infection	II	1	Wound infection	III	1
			Pneumonia	II	2
			Pleuritis	II	1
			Heart failure	II	1

BPF, bronchopleural fistula; AE, acute exacerbation

**Fig. 2:**
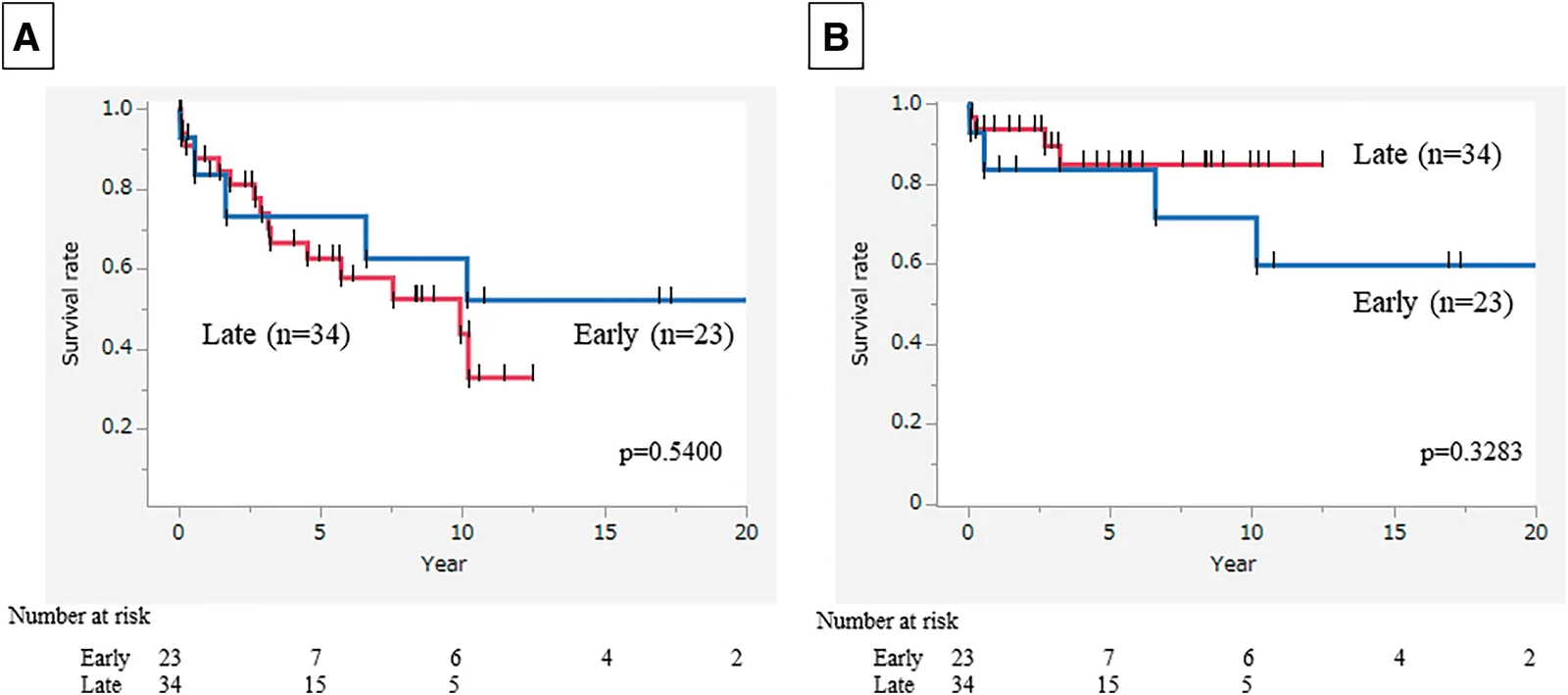
Survival outcomes according to treatment era. (**A**) Overall survival (OS) stratified by early and late treatment periods. (**B**) Disease-specific survival (DSS) stratified by early and late treatment periods.

**Fig. 3:**
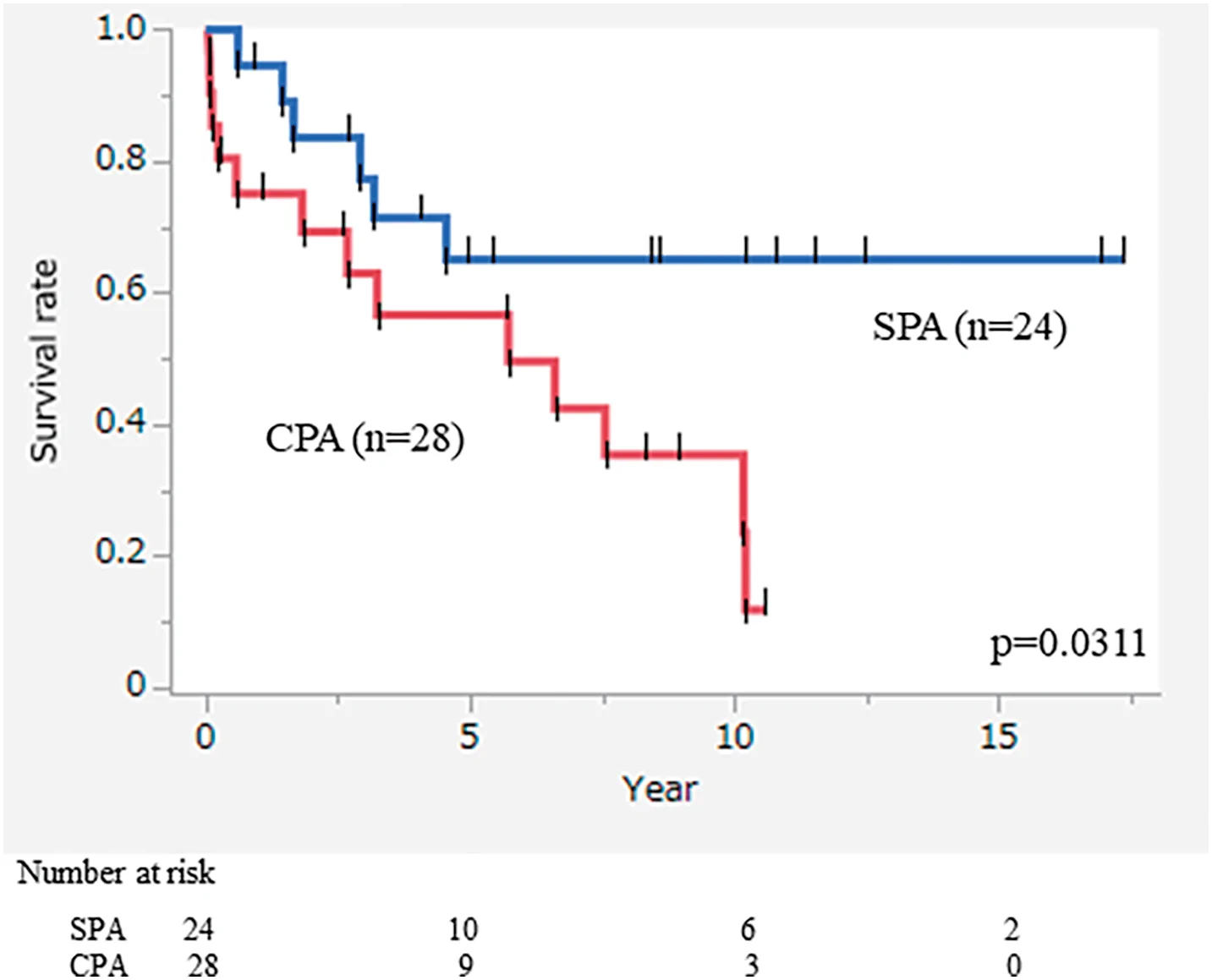
Comparison of overall survival between simple and chronic pulmonary aspergillosis. Kaplan–Meier curves demonstrating overall survival in patients with simple pulmonary aspergillosis (SPA) and chronic pulmonary aspergillosis (CPA).

The median follow-up period was 5.1 years. OS (log-rank p = 0.5400; HR 0.724, 95% CI 0.26–2.04) and disease-specific survival (log-rank p = 0.3283; HR 2.29, 95% CI 0.55–9.34) did not significantly differ between the early and late eras (**Fig. 2**). However, patients with CPA demonstrated significantly worse OS than those with SPA (log-rank p = 0.0311; HR 0.358, 95% CI 0.13–0.95) (**Fig. 3**). Preoperative (log-rank p= 0.1622; HR 0.520, 95% CI 0.20–1.32) and postoperative (log-rank p = 0.8013; HR 1.127, 95% CI 0.44–2.86) antifungal therapy did not significantly affect OS. Although the additional analysis was limited to patients with SPA, neither preoperative nor postoperative antifungal administration was associated with a significant difference in OS (p = 0.2357 and p = 0.3554, respectively).

## Discussion

The present study demonstrated significant improvements in perioperative outcomes of surgery for pulmonary aspergillosis over a 30-year period, with reduced operative time, intraoperative blood loss, and postoperative hospital stay. Despite these advances, major postoperative complications and long-term survival remained largely unchanged. These findings indicate that while refinements in perioperative management and surgical techniques have improved operative efficiency, long-term outcomes might be influenced by the type of aspergillosis, underlying pulmonary disease and patient comorbidities.

Pulmonary aspergillosis remains one of the most technically demanding benign thoracic diseases. Chronic inflammation, dense pleural adhesions, hilar fibrosis, and developed collateral vessels frequently result in difficult hilar dissection and massive bleeding. Previous reports have demonstrated postoperative complication rates ranging from 20% to 40% and mortality rates of 4%–15%, particularly in patients with complex aspergillomas or CPA.^6–14)^ Our perioperative outcomes (major complication rate, 26%; surgical mortality, 3.5%) were comparable with those reported previously. Importantly, major postoperative complications remained unchanged despite significant reductions in operative time and blood loss. This may partly reflect the increasing complexity of surgical candidates, as chronic obstructive pulmonary disease (COPD) and interstitial pneumonia were significantly more common in the late era. Further improvement therefore may require not only technical advances but also careful patient selection and perioperative optimization.

Several strategies may contribute to reducing postoperative morbidity. First, careful patient selection and appropriate timing of surgery are essential. In patients with CPA, surgery may be considered before extensive parenchymal destruction develops, when disease is localized and resectable, cardiopulmonary reserve is adequate, and clinically significant symptoms or radiological progression persist despite appropriate medical therapy. Timely multidisciplinary assessment may therefore help identify an appropriate surgical window before resection becomes excessively invasive. Second, the extent of lung resection should be carefully determined according to disease extent and pulmonary reserve, together with meticulous surgical techniques, including careful hilar dissection and reinforcement of the bronchial stump using vascularized tissue in high-risk patients. Last, perioperative nutritional and respiratory optimization may be important because many patients have chronic inflammation, cachexia, and poor pulmonary function.

BAE remains an important adjunctive strategy in patients with active hemoptysis.^22,23)^ Massive hemoptysis is one of the most life-threatening manifestations of pulmonary aspergillosis, and urgent bleeding control is occasionally necessary before definitive surgery. BAE can stabilize respiratory status and reduce the immediate risk of fatal hemorrhage, functioning as an effective bridge-to-surgery approach.^14)^ However, the role of BAE might remain controversial. In the present study, patients who underwent BAE experienced greater intraoperative blood loss and longer operative time. This likely reflects selection bias because BAE was preferentially used in patients with advanced inflammatory disease and severe hemoptysis. In addition, repeated BAE may promote hilar fibrosis and tissue fragility, potentially increasing surgical difficulty. Furthermore, although BAE is effective for temporary bleeding control, recurrence of hemoptysis is common without definitive treatment. Therefore, surgery should still be considered in selected patients after stabilization with BAE.

The development of antifungal therapy has also substantially changed the management of pulmonary aspergillosis.^24–27)^ Since the introduction of triazole agents such as itraconazole and voriconazole, outcomes of medical treatment for CPA have improved considerably. Antifungal therapy may suppress inflammatory activity, reduce fungal burden, and improve general condition before surgery. Postoperatively, antifungal treatment may also reduce recurrence risk in patients with residual lesions or bilateral disease. Nevertheless, in the present study, neither preoperative nor postoperative antifungal therapy significantly improved OS. This finding should be interpreted cautiously because treatment was not standardized over the 30-year study period and was influenced by disease severity and changes in available antifungal agents, including the use of fluconazole in the earlier era. Selection bias and confounding by indication are therefore unavoidable. Moreover, long-term prognosis, particularly in patients with CPA, may be strongly influenced by underlying pulmonary disease and frailty.^28)^ Thus, our findings should not be interpreted as evidence against the benefit of contemporary antifungal therapy. Mold-active antifungal therapy remains an important component of multidisciplinary management, particularly for CPA or residual disease, and should be individualized according to disease status and completeness of resection. Notably, a Japanese national database study demonstrated that preoperative antifungal therapy was associated with reduced postoperative complications after pulmonary resection for pulmonary aspergillosis.^24)^

Interestingly, despite significant improvements in perioperative outcomes, OS did not improve in the late era. At first glance, this finding appears paradoxical because patients in the late era had fewer symptoms at presentation and comparable BMI. However, these conventional clinical variables do not necessarily reflect the complexity of patients’ underlying conditions. Over the past 3 decades, the clinical background of pulmonary aspergillosis has changed substantially. In the early era, most patients developed aspergillosis in post-tuberculosis destroyed lungs, whereas patients in the late era increasingly had comorbidities, such as COPD, interstitial lung disease, and cardiovascular and renal diseases. These underlying comorbidities, rather than the aspergillosis itself, may have exerted a greater influence on long-term survival despite improvements in perioperative management. In addition, CPA itself was associated with significantly worse survival than SPA in our cohort, consistent with previous reports demonstrating the progressive and systemic nature of CPA.^5)^ Therefore, improvements in operative safety alone may be insufficient to improve long-term survival.

To further improve long-term outcomes, perioperative pulmonary rehabilitation, nutritional support, smoking cessation, and optimization of chronic respiratory disease may contribute to improved postoperative recovery and long-term survival. Long-term multidisciplinary follow-up after surgery is also essential because recurrence, contralateral disease progression, and chronic respiratory failure may occur even after successful local treatment.

The marked reductions in operative time and blood loss in the late era are unlikely to be attributable to VATS alone, because minimally invasive surgery was performed in only a limited proportion of patients. These improvements may also reflect cumulative advances in surgical practice over the 3 decades, including refinements in hilar dissection and hemostasis, improved management of dense adhesions and collateral vessels, and advances in surgical devices. Improvements in perioperative and multidisciplinary management may also have contributed to better operative efficiency and recovery.

Minimally invasive surgery may represent another promising strategy for reducing surgical invasiveness in selected patients. More recently, robotic surgery has attracted increasing attention because of its superior visualization, articulated instruments, and improved dexterity in complex hilar dissection. Although evidence regarding robotic surgery for pulmonary aspergillosis remains limited, robotic approaches may facilitate safer minimally invasive surgery in selected patients with localized disease.^20)^ Future accumulation of experience and comparative studies will be necessary to define the role of robotic surgery in infectious thoracic diseases.

This study has several limitations. First, it was a retrospective single-institution study with a relatively small sample size. Second, selection bias was unavoidable regarding indications for surgery, BAE, and antifungal therapy. Third, detailed analyses of pulmonary function and postoperative quality of life were not available. A final limitation of this study was the nonuniform duration and completeness of follow-up. Because this retrospective study spanned more than 3 decades, some patients were lost to long-term follow-up after referral to local hospitals or discontinuation of outpatient visits. Therefore, comparisons of long-term survival between the 2 eras should be interpreted with caution. Nevertheless, this study demonstrates that surgery for pulmonary aspergillosis can be performed with acceptable perioperative outcomes in appropriately selected patients and provides practical information for contemporary multidisciplinary management. Future multicenter collaborative studies and nationwide databases will be necessary to establish optimal indications, perioperative strategies, and minimally invasive approaches for this challenging disease.

## Conclusions

Perioperative outcomes of surgical treatment for pulmonary aspergillosis improved over the past 3 decades, with significant reductions in operative time, blood loss, and postoperative hospital stay, while major postoperative complications and long-term survival remained unchanged. Patients with CPA had significantly poorer long-term survival than those with SPA. Careful patient selection and multidisciplinary perioperative management remain essential, and further multicenter studies are warranted to optimize surgical strategies for this challenging disease.

## Declarations

### Data availability

The data that support the findings of this study are available from the corresponding author upon reasonable request.
